# Gomisin A Suppresses Colorectal Lung Metastasis by Inducing AMPK/p38-Mediated Apoptosis and Decreasing Metastatic Abilities of Colorectal Cancer Cells

**DOI:** 10.3389/fphar.2018.00986

**Published:** 2018-08-29

**Authors:** Ji-Ye Kee, Yo-Han Han, Jeong-Geon Mun, Seong-Hwan Park, Hee D. Jeon, Seung-Heon Hong

**Affiliations:** Department of Oriental Pharmacy, College of Pharmacy, Wonkwang-Oriental Medicines Research Institute, Wonkwang University, Iksan, South Korea

**Keywords:** AMPK, apoptosis, colorectal cancer, gomisin A, lung metastasis, p38

## Abstract

Gomisin A (G.A) is a dietary lignan compound from *Schisandra chinensis*. In this study, the effect of G.A on the proliferation and metastasis of colorectal cancer (CRC) cells was investigated using several CRC cell lines and a lung metastasis mouse model. Both oral and intraperitoneal administration of G.A (50 mg/kg) inhibited lung metastasis of CT26 cells. Various concentrations of G.A were incubated with CRC cell lines and their viability was determined using a cell counting kit-8 assay. G.A significantly decreased the viability of various CRC cell lines, whereas it did not change the proliferation of normal colon cells. G.A induced G0/G1 phase arrest and apoptosis of CT26 and HT29 cells by regulating cyclin D1/cyclin-dependent kinase 4 (CDK4) expression and apoptotic proteins such as caspases and B-cell lymphoma-2 (Bcl-2) family proteins, respectively. G.A-induced apoptosis was mediated by AMPK/p38 activation in CRC cells. A non-cytotoxic concentration of G.A inhibited epithelial–mesenchymal transition of CRC cells by modulating E-cadherin and N-cadherin expression levels. Moreover, the migration and invasion of CRC cells were reduced by G.A treatment. Especially, G.A decreased matrix metalloproteinase (MMP)-2 and MMP-9 expressions and activities. G.A ameliorated lung metastasis of CRC cells by decreasing cell survival and metastatic abilities of CRC cells. Thus, G.A might be a potential novel therapeutic agent for metastatic CRC.

## Introduction

Colorectal cancer (CRC) is the most frequently diagnosed cancer and one of the main causes of cancer mortality worldwide (Miller et al., 2016). CRC progression results in the spread of cancer cells to distant organs such as the liver, lung, brain, and bone, which leads to metastatic cancer (Patanaphan and Salazar, 1993). Metastasis is composed of several cellular processes including the development of a metastatic phenotype by cancer cells, movement of cells through the blood and lymphatic vessels, invasion of cancer cells into target tissues, and colonization. Since the high mortality of CRC is related to metastasis (Klein, 2008; Siegel et al., 2017), its prevention and treatment in CRC could improve the prognosis of patients with CRC.

Metastasis is initiated by the migration of cancer cells through circulating blood and lymphatic vessels, and then these cells invade distant organs (Martin et al., 2013). Epithelial–mesenchymal transition (EMT) is one of the important cellular process for the acquisition of metastatic phenotypes including migration and invasion by cancer cells (Kalluri and Weinberg, 2009). A typical feature of EMT is the loss of cell-cell contact by the regulation of expression of cell-cell adhesion molecules such as E-cadherin, N-cadherin, and vimentin (Thiery and Sleeman, 2006). Additionally, epithelial cancer cells become spindle shaped with increased metastatic abilities including migration and invasion by enhancing matrix metalloproteinase (MMP)-2 and MMP-9 production, which are involved in matrix remodeling-related proteolysis (Khasigov et al., 2003).

AMP-dependent protein kinase (AMPK) is a serine-threonine kinase that participates in an energy-sensing cascade related to cellular homeostasis (Hardie et al., 2012). In various types of cancer cells, activation of AMPK induces apoptosis via the p38, Akt, and mechanistic target of rapamycin (mTOR) signaling pathway, which could regulate p21, p27, and p53 expression; B-cell lymphoma-2 (Bcl-2) family proteins, and cleavage of caspases (Li et al., 2015). Previous studies have reported that the AMPK activator AICAR decreased the viability of human CRC cells, whereas the AMPK inhibitor compound C (CC) increased the proliferation of CRC cells (Lea et al., 2014). Moreover, several natural products and active compounds such as *Torilis japonica, Oldenlandia diffusa*, quercetin, and kazinol C induced apoptosis of CRC cells by AMPK activation (Kim et al., 2010, 2015, 2016; Lu et al., 2016).

The fruit of *Schisandra chinensis* (Schisandrae Fructus) is cultivated in East Asia and it has been used as a beverage, wine, and tea (Szopa et al., 2017). Its Korean name “Omija” (Gomishi in Japanese and Wu-wei-zi in Chinese) is derived from its interesting characteristic of five different tastes in the fruit: sour (fruit skin), sweet (flesh), bitter/spicy (seed), and salty (all parts) (Panossian and Wikman, 2008). In traditional medicine, this fruit has been widely used to treat hepatitis, cough, spontaneous sweating, diabetes, heart disease, rheumatoid arthritis, skin irritation, and insomnia (Panossian and Wikman, 2008; Szopa et al., 2017). According to previous studies, many kinds of lignans such as gomisin A (G.A), gomisin B, gomisin C, gomisin G, gomisin N, and schisandrin have been isolated from Schisandrae Fructus as major constituents (Panossian and Wikman, 2008; Szopa et al., 2017). G.A has multiple biological activities including memory improving and hepatoprotective, antihypertensive, antidiabetic, and anti-inflammatory effects (Kim et al., 2006; Kwon et al., 2011; Park et al., 2012; Wang et al., 2014; Jiang et al., 2015). G.A also inhibits hepatocarcinogenesis and shows cytotoxicity against human CRC cell lines (LoVo, HCT116) and A2780 human ovarian cancer cells (Ohtaki et al., 1996; Smejkal et al., 2010; Hwang et al., 2011; Jeong et al., 2017). However, the effects of G.A on the metastatic phenotype and metastasis of CRC cells have not been elucidated using *in vivo* models.

In this investigation, the effects of G.A on CT26, MC38, HT29, and SW620 CRC cell lines were explored, including cell cycle arrest, apoptosis, and the related signaling pathways. Typical metastatic phenotypes such as EMT, migration, and invasion of CRC cells were evaluated after G.A treatment. Moreover, the antimetastatic effects of G.A on CRC cells were confirmed using a lung metastasis mouse model.

## Materials and Methods

### Reagents and Cell Lines

Anti-phospho-AMPK, phospho-p38, phospho-ERK, phospho-JNK, phospho-Akt, AMPK, poly (ADP-ribose) polymerase (PARP), caspase-3, caspase-9, Bcl-2, Bcl-extra-large (Bcl-xL), and Bcl-2-associated X protein (Bax) antibodies (Cell Signaling, Danvers, MA, United States). Anti-p38, ERK, JNK, Akt, γH2AX, β-actin, and α-tubulin antibodies were purchased from Santa Cruz Biotechnology (Santa Cruz, CA, United States). SB203580 was obtained from Sigma-Aldrich (St. Louis, MO, United States). Compound C (CC) was purchased from MedChemExpress (Monmouth Junction, NJ, United States). Matrigel was obtained from BD Biosciences (San Diego, CA, United States). The cell counting kit (CCK)-8 was purchased from Enzo Life Sciences (Farmingdale, NY, United States). The mouse CRC cell line CT26 and MC38, human CRC cell line HT29 and SW620, and normal CCD-18co colon cell line were purchased from Korean Cell Line Bank (Seoul, South Korea) and maintained in Dulbecco’s modified Eagle’s medium (DMEM) and Roswell Park Memorial Institute (RPMI) 1640 at 37°C in a 5% CO_2_ incubator.

### Animals

Female BALB/c mice (5-week-old) were purchased from Samtaco Korea (Osan, South Korea). The mice were housed individually in ventilated cages in a laminar air-flow room. All animal experimental protocols, care, and handling were approved by Wonkwang University Institutional Animal Care and Use Committee (IACUCs, WKU 17-91).

### *In vivo* Model of Lung Metastasis

To establish the experimental lung metastasis model, 2 × 10^5^ cells were injected into the tail vein of mice intravenously (i.v.). The mice were orally or intraperitoneally administered 50 mg/kg G.A 2 h prior to the injection of CT26 cells and were subsequently euthanized 14 days later, and the lungs were harvested and stained with Bouin’s solution. The number of all tumor colonies in the lung was counted to evaluate the antimetastatic effect of G.A.

### Cell Viability Assay

The viability of G.A-treated cells was measured using the CCK-8 assay. Briefly, 3 × 10^3^ cells/well were plated in a culture plate treated with G.A for 72 h. The medium was changed to the fresh medium containing the CCK-8 reagent, and the absorbance was determined at 450 nm using a microplate reader.

### Cell Cycle Analysis

Cells were plated in 6-well plates (1 × 10^6^ cells/well) and treated with G.A (0–100 μM) for 24 h. The cell cycle distribution was determined using the Muse cell cycle kit (Millipore, Bedford, MA, United States) according to the manufacturer’s protocols. The cells were stained with cell cycle reagent and analyzed using a Muse cell analyzer (MUSE, Millipore, Bedford, MA, United States).

### Real-Time Reverse Transcription-Polymerase Chain Reaction (RT-PCR)

Total RNA was isolated from cells and tissues using an RNA-spin™ total RNA extraction kit (iNtRon Biotech, Seoul, South Korea) and reverse transcribed to cDNA using the Power cDNA synthesis kit (iNtRon Biotech, Seoul, South Korea). The real-time reverse transcription-polymerase chain reaction (RT-PCR) was carried out using the Power SYBR^®^ Green PCR Master Mix and Step-one Plus™ real-time PCR systems (Applied Biosystems, Foster City, CA, United States). The primer sequences are described in **Table 1**.

**Table 1:** Primer sequences for the Real-time RT-PCR.

Genes	Primer sequences (5′–3′)
Mouse STAT1	TGGTGAAATTGCAAGAGCTG (Forward)
	CAGACTTCCGTTGGTGGATT (Reverse)
Mouse E-cadherin	AATGGCGGCAATGCAATCCCAAGA (Forward)
	TGCCACAGACCGATTGTGGAGATA (Reverse)
Mouse N-cadherin	TGGAGAACCCCATTGACATT (Forward)
	TGATCCCTCAGGAACTGTCC (Reverse)
Mouse Vimentin	CGGAAAGTGGAATCCTTGCA (Forward)
	CACATCGATCTGGACATGCTG (Reverse)
Mouse Cyclin D1	TAGGCCCTCAGCCTCACTC (Forward)
	CCACCCCTGGGATAAAGCAC (Reverse)
Mouse CDK4	AGAGCTCTTAGCCGAGCGTA (Forward)
	TTCAGCCACGGGTTCATATC (Reverse)
Mouse GAPDH	GACATGCCGCCTGGAGAAAC (Forward)
	AGCCCAGGATGCCCTTTAGT (Reverse)
Human STAT1	CTAGTGGAGTGGAAGCGGAG (Forward)
	CACCACAAACGAGCTCTGAA (Reverse)
Human E-cadherin	GTCAGTTCAGACTCCAGCCC (Forward)
	AAATTCACTCTGCCCAGGACG (Reverse)
Human N-cadherin	CTCCATGTGCCGGATAGC (Forward)
	CGATTTCACCAGAAGCCTCTAC (Reverse)
Human Vimentin	TCTACGAGGAGGAGATGCGG (Forward)
	GGTCAAGACGTGCCAGAGAC (Reverse)
Human Cyclin D1	ATGCCAACCTCCTCAACGAC (Forward)
	GGCTCTTTTTCACGGGCTCC (Reverse)
Human CDK4	GTGCAGTCGGTGGTACCTG (Forward)
	TTCGCTTGTGTGGGTTAAAA (Reverse)
Human GAPDH	TGCACCACCACCTGCTTAGC (Forward)
	GGCATGGACTGTGGTCATGAG (Reverse)

### Apoptosis Assay

Cell apoptosis was determined using the terminal deoxynucleotidyl transferase (TdT) deoxyuridine dUTP nick-end labeling (TUNEL) and Annexin V assays as previously described (Kee et al., 2017). CT26 and HT29 cells were seeded in six-well plates, treated with G.A for 24 h, fixed with 3.7% paraformaldehyde for 20 min, and then washed with phosphate-buffered saline (PBS). The cells were incubated with 4′,6-diamidino-2-phenylindole (DAPI) solution (2.5 μg/mL) for 3 min at room temperature and washed with PBS. Apoptotic cells and stained nuclei were observed using a fluorescence microscope (Carl Zeiss, Oberkochen, Germany). In addition, the apoptotic cells were analyzed using the MUSE Annexin V and dead cell kit (Millipore, Billerica, MA, United States) in accordance with the recommended protocol. The stained cells were analyzed using the Muse cell analyzer.

### Western Blot Analysis

Proteins from metastatic lung tissues and G.A-treated cells were lysed using PRO-PREP™ protein extraction solution (iNtRon Biotech, Seoul, South Korea). Samples were centrifuged for 10 min, and the supernatant was mixed with 2× sample buffer after quantifying the protein using the bicinchoninic acid (BCA) protein assay. Total proteins were separated using gel electrophoresis and transferred onto membranes. Specific proteins were detected with the primary antibodies and horseradish peroxidase-conjugated antibodies (Dako, Glostrup, Denmark). Protein bands were detected using and FluorChem M system (ProteinSimple, San Jose, CA, United States).

### Immunofluorescence (IF)

Cells were seeded in an eight-well chamber slide (2 × 10^3^ cells/well), stabilized, treated with G.A for 24 h, fixed, and then blocked with 3% bovine serum albumin (BSA). The cells were incubated with primary antibodies against E-cadherin, N-cadherin, MMP-2, and MMP-9 overnight. Alexa Fluor antibodies were used as the secondary antibodies, and then the cells were stained with DAPI. Images were viewed using a Zeiss Observer.A1 microscope (Carl Zeiss, Germany).

### Migration and Invasion Assay

Cells were seeded in a six-well plate, allowed to reach 90% confluence, and then they were scratched using a 200 μL pipet tip. After washing with PBS, serum-free medium containing G.A was added to the cells, followed by 24 h incubation. The scratches were monitored, and images were acquired using an EVOS microscope (Thermo Fisher Scientific, Waltham, MA, United States).

Invasion ability was evaluated using a matrigel-coated transwell chamber. Cells (2 × 10^4^ cells/mL) were seeded in the upper part of the transwell chamber with serum-free medium with or without G.A. The lower chamber was filled with 10% fetal bovine serum (FBS)-containing medium. After incubation for 24 h, cells on the inner side of the chambers were fixed with 3.7% paraformaldehyde (5 min), treated with 100% methanol (20 min), and stained with Giemsa (15 min). The chamber was dried, and the stained cells were photographed under a microscope (EVOS, Thermo Fisher Scientific, Waltham, MA, United States).

### Gelatin Zymography

Matrix metalloproteinase activity was determined using a Zymogram buffer Kit (Komabiotech, Seoul, South Korea). G.A was added to 5 × 10^5^ cells/well in six-well plates and incubated for 24 h. The supernatant was collected and mixed with zymogram sample buffer. After electrophoresis using a 0.1% acrylamide gel, the gel was incubated in zymogram renaturing buffer for 30 min, followed by incubation in zymogram developing buffer for 30 min. Coomassie blue R-250 solution was used to visualize the gelatinolytic activity of the MMPs after incubation at 37°C for 24 h.

### Statistical Analysis

The data are the means ± standard deviation (SD) of a minimum of three experiments and statistically significant differences were analyzed using the Student’s *t*-test. *p*-Value < 0.05 was considered statistically significant.

## Results

### Gomisin A Suppresses Lung Metastasis of CT26 Cells

**FIGURE 1:**
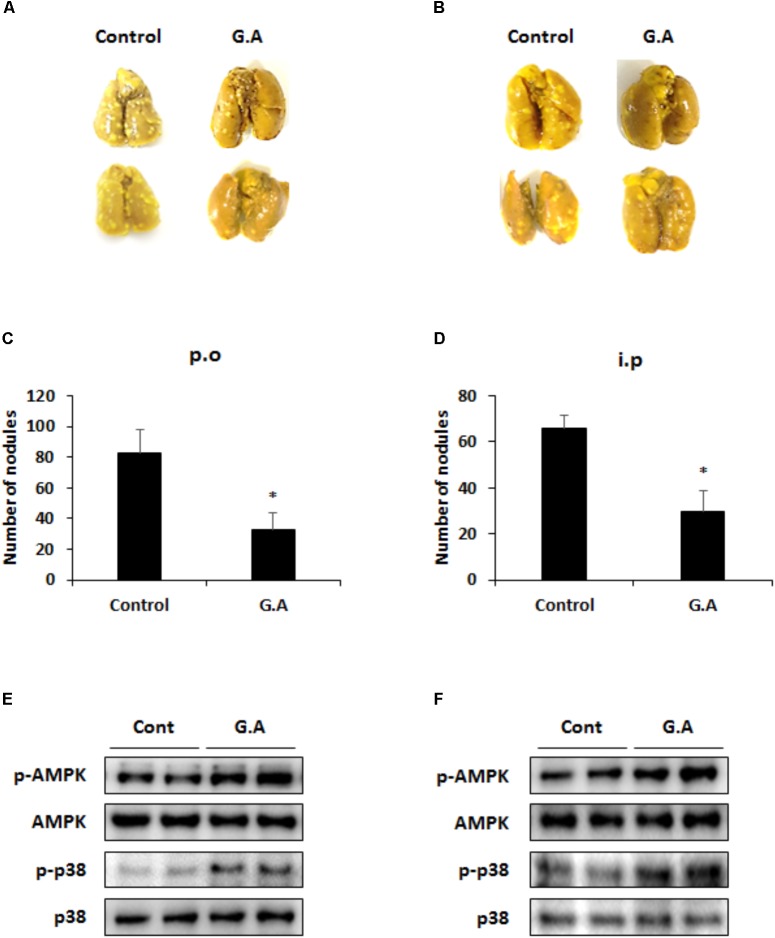
Effect of gomisin A (G.A) administration on lung metastasis of CT26 cells. Lung tissues were excised, and nodules were counted to evaluate antimetastatic effect of orally administered G.A **(A,C)** and intraperitoneal injection **(B,D)**. Phosphorylation of AMPK and p38 in metastatic lung tissues after oral administration **(E)** and intraperitoneal injection **(F)**. Data are means ± standard deviation (SD) of three independent experiments; ^∗^*p* < 0.05.

At the end of the *in vivo* experiment, the mouse lungs were excised to investigate the effect of G.A on the metastatic CRC and serum samples were collected to evaluate the toxicity of G.A in the mice. The body weight and parameters of liver and kidney function of the G.A-treated group of mice did not significantly differ from those of the control group mice (**Table 2**). After 14 days of G.A administration, the lung metastasis of CT26 cells was clearly inhibited (**Figures 1A,B**). The number of nodules in the lung tissues was 32.75 ± 10.71 and 82.5 ± 15.06 in the G.A orally-administered and control mice, respectively (**Figure 1C**). Moreover, intraperitoneal administration of G.A decreased the number of nodules in lung tissues from 66 ± 5.35 to 29.75 ± 8.92 (**Figure 1D**). In the lung metastatic tissues, AMPK and p38 phosphorylation were also increased by G.A administration (**Figures 1E,F**). Based on these results, G.A inhibited the metastasis of CRC cells by activating AMPK and p38.

**Table 2:** Body weight and serum parameters of gomisin A (G.A)-treated CT26-injected mice.

Treatment		B.W (g)	AST (S)	ALT (S)	Creatinine (S)	BUN (S)
p.o	Cont	18.27 ± 0.59	98 ± 28.24	45 ± 28.24	0.12 ± 0.01	25 ± 0.7
	G.A	18.25 ± 0.75	80 ± 19.73	39 ± 42.43	0.12 ± 0.04	28 ± 2.12
i.p	Cont	18.42 ± 1.07	191 ± 27.07	102 ± 15.55	0.15 ± 0.03	28 ± 4.24
	G.A	18.38 ± 0.49	192 ± 13.28	80 ± 6.42	0.18 ± 0.01	24 ± 1.15

*p.o., Oral administration; i.p., intraperitoneal injection; B.W., body weight; AST, aspartate transaminase; ALT alanine transaminase; BUN, blood urea nitrogen.*

### Gomisin A Inhibits CRC Cell Survival

**FIGURE 2:**
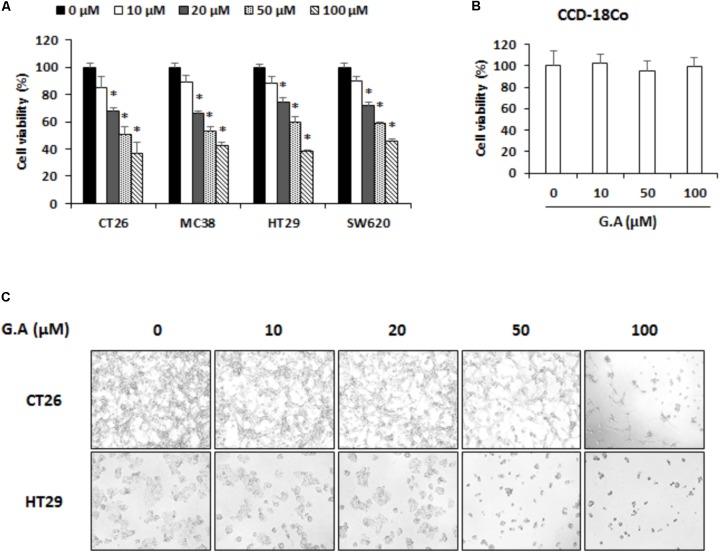
Effect of gomisin A (G.A) on viability of colorectal cancer (CRC) cells. **(A)** CRC cell lines CT26, MC38, HT29, and SW620 and **(B)** normal CCD-18Co colon cell lines were seeded in 96-well plates and treated with G.A (0–100 μM) for 48 h. Cell viability was evaluated using cell counting kit (CCK)-8 assay. **(C)** Morphological changes of G.A-treated CT26 and HT29 cells. Images were acquired using phase contrast microscope (magnification 200×). Data are means ± standard deviation (SD) of three independent experiments; ^∗^*p* < 0.05.

To confirm the cytotoxicity of G.A on the various CRC cells, murine CRC cell lines, CT26 MC38, and human CRC cell lines, HT29 and SW620, were treated with for 48 h. As shown in **Figure 2A**, 20–100 μM but not 10 μM G.A concentration-dependently decreased the viability of CRC cells. The same concentrations of G.A did not change the proliferation of the normal CCD-18Co colon cell line (**Figure 2B**). After 72 h incubation with G.A, morphological changes, including floating cells, shrunken, and round-shaped cells, were observed in CT26 and HT29 cells treated with higher concentrations of G.A (50 and 100 μM, **Figure 2C**).

### Gomisin A Induces G0/G1 Phase Arrest of CRC Cells

**FIGURE 3:**
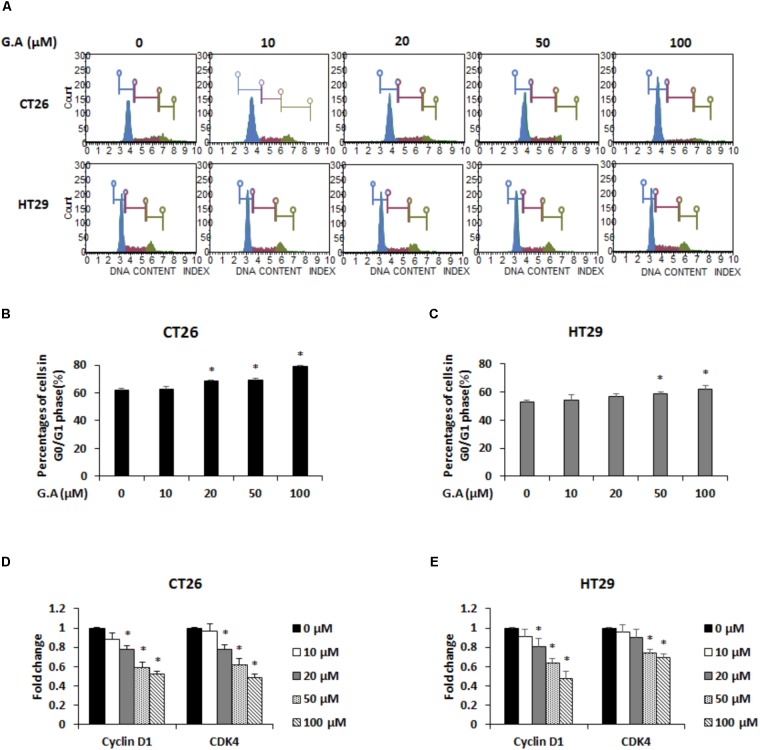
Effect of gomisin A (G.A) on cell cycle distribution of CRC cells. **(A)** CT26 and HT29 cells were treated with G.A (0–100 μM) for 24 h. Cell cycle analysis was conducted using Muse cell cycle kit. **(B,C)** G0/G1 phase cells (%) were determined using flow cytometry. **(D,E)** Expression levels of cyclin D1 and cyclin-dependent kinase 4 (CDK4) in CT26 **(D)** and HT29 **(E)** cells measured using real-time reverse transcription-polymerase chain reaction (RT-PCR). Data are means ± standard deviation (SD) of three independent experiments; ^∗^*p* < 0.05.

To assess the effect of G.A on the cell cycle phase distributions of CRC cells, after G.A treatment for 24 h, the cells were propidium iodide (PI)-stained and analyzed using flow cytometry. High concentration of G.A (50 and 100 μM) increased the percentages of cells in the G0/G1 phase (**Figures 3A–C**). The cyclin/cyclin-dependent kinase (CDK) complex controls the cell cycle phases (Lim and Kaldis, 2013). Since cyclin D1 and CDK4 are associated with the progression of G1 phase cells to the S phase, mRNA expression levels of cyclin D1 and CDK4 were determined using real-time RT-PCR. G.A significantly downregulated the cyclin D1 and CDK4 expression levels in CT26 and HT29 cells (**Figures 3D,E**). According to previous study, G.A can induce cell cycle arrest of HeLa cells by regulating cyclin D1 expression via STAT1 signaling (Waiwut et al., 2012). Thus, mRNA expression levels of STAT1 in G.A-treated CT26 and HT29 cells were confirmed and high concentration of G.A significantly decreased STAT1 expression (**Supplementary Figure 1**). These results indicate that G.A might cause G0/G1 phase cell cycle arrest by regulating the cyclin D1-CDK4 complex.

### Gomisin A Promotes Apoptosis of CRC Cells by Inducing AMPK and p38 Phosphorylation

**FIGURE 4:**
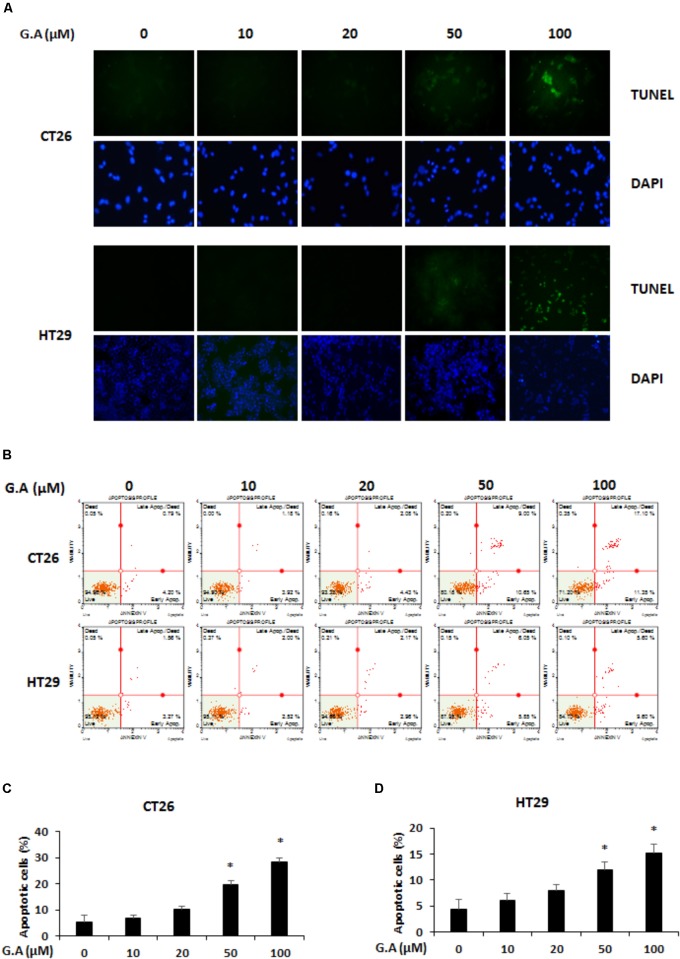
Effect of gomisin A (G.A) on apoptosis of CRC cells. **(A)** CT26 and HT29 cells were treated with G.A (0–100 μM) for 24 h, and apoptotic cells were detected using terminal deoxynucleotidyl transferase (TdT) deoxyuridine dUTP nick-end labeling (TUNEL) assay. Images were captured using a phase contrast microscope (magnification 200×). **(B)** G.A-treated CT26 and HT29 cells were analyzed using Annexin V assay. **(C,D)** Percentage of apoptotic CT26 **(C)** and HT29 **(D)** cells. Data are means ± standard deviation (SD) of three independent experiments; ^∗^*p* < 0.05.

**FIGURE 5:**
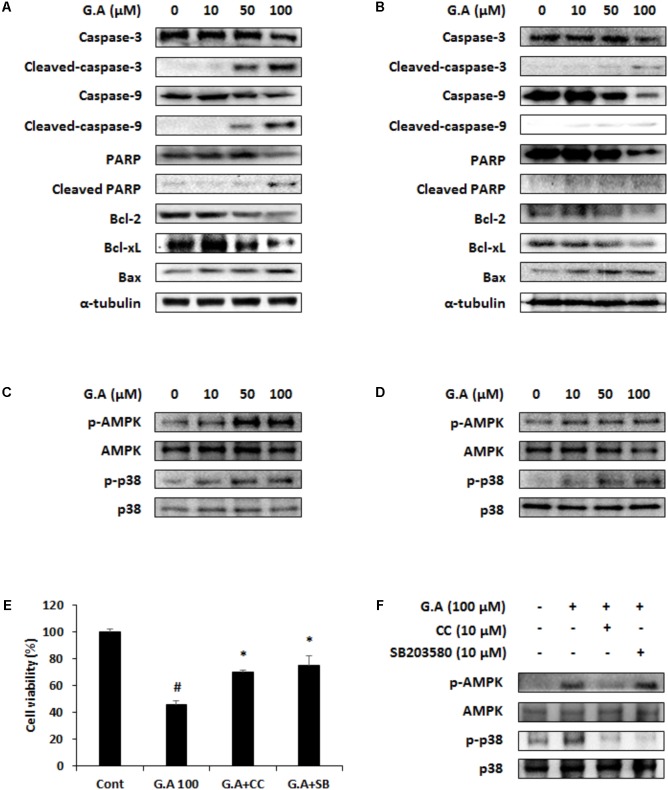
Effect of gomisin A (G.A) on AMP-dependent protein kinase (AMPK) and p38 phosphorylation in CRC cells. **(A,B)** Apoptotic proteins were detected using western blot analysis in CT26 **(A)** and HT29 **(B)** cells. **(C,D)** Phosphorylation of AMPK and p38 was detected using western blot analysis in CT26 **(C)** and HT29 **(D)** cells. **(E)** Cell viability was measured in G.A-treated CT26 cells after CC and SB203580 treatment for 24 h. **(F)** AMPK and p38 phosphorylation was confirmed after CC and SB203580 treatment of G.A-treated CT26 cells. Data are means ± standard deviation (SD) of three independent experiments; ^#^*p* < 0.05 versus control and ^∗^*p* < 0.05 versus G.A alone.

We investigated whether G.A could induce apoptosis of CRC cells using TUNEL and Annexin V assays. The results showed that 50 and 100 μM G.A treatment increased the TUNEL- and Annexin V-positive (**Figures 4A,B**, respectively) CT26 and HT29 cells. According to the Annexin V assay result, apoptotic CT26 and HT29 cells were significantly increased by G.A treatment for 24 h (**Figures 4C,D**). Moreover, cleavage of caspase-9, caspase-3, and PARP was induced by G.A treatment while Bcl-2 and Bcl-xL were downregulated and Bax was upregulated in CT26 and HT29 cells (**Figures 5A,B**). AMPK and mitogen-activated protein kinase (MAPK) activity has been recognized to be related to the proliferation of CRC cells (Lea et al., 2014). Thus, we investigated whether G.A activates AMPK and MAPKs including extracellular signal-regulated kinase (ERK), c-Jun N-terminal kinase (JNK), and p38.

The cells treated with G.A showed increased phosphorylation levels of AMPK and p38 (**Figures 5C,D**), whereas ERK, JNK, and Akt activation was not changed by G.A treatment (**Supplementary Figure 2**). Then, we investigated whether blockade of AMPK and p38 activation could suppress the G.A-induced reduction in viability of CRC cells. CT26 cells were treated with compound C (10 μM) and SB203580 (10 μM), which are AMPK and p38 selective inhibitors, respectively. Blocking AMPK and p38 phosphorylation partially recovered the viability of G.A-treated CT26 cells (**Figure 5E**). Additionally, CC decreased both AMPK and p38 phosphorylation induced by G.A treatment, whereas SB203580 inhibited only the phosphorylation of p38 in CT26 cells (**Figure 5F**). These findings suggest that the G.A-induced apoptosis was likely mediated via the AMPK/p38 signaling pathway.

### Gomisin A Suppresses EMT of CRC Cells

**FIGURE 6:**
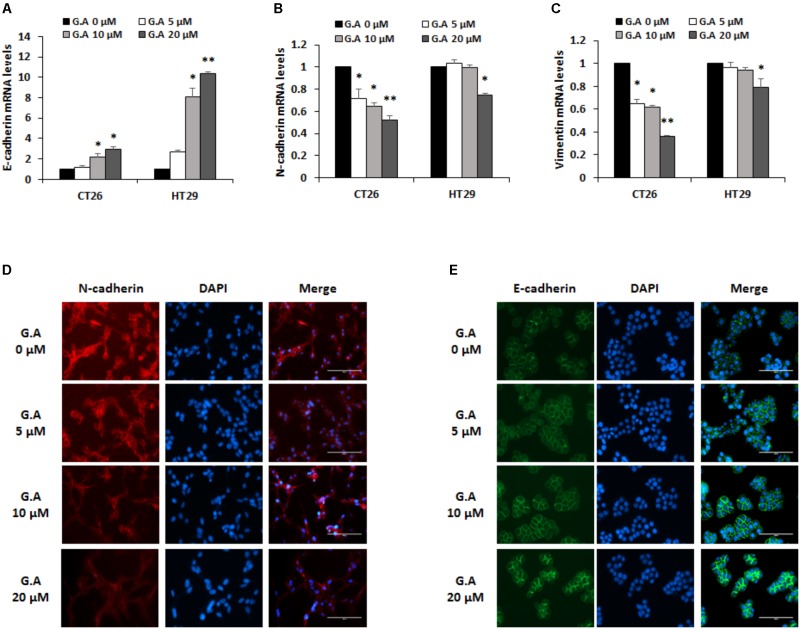
Effect of gomisin A (G.A) on the EMT markers of CRC cells. CT26 and HT29 cells were treated with G.A (0–20 μM) for 24 h, and expression levels of epithelial–mesenchymal transition (EMT) markers were evaluated. **(A–C)** EMT-related markers E-cadherin **(A)**, N-cadherin **(B)**, and vimentin **(C)** were measured using real-time reverse transcription-polymerase chain reaction (RT-PCR); ^∗^*p* < 0.05 and ^∗∗^*p* < 0.01. **(D)** Expression of N-cadherin in G.A-treated CT26 cells. **(E)** Expression of E-cadherin in G.A-treated HT29 cells. Images were captured using phase contrast microscope (magnification 200×). Data are means ± standard deviation (SD) of three independent experiments.

EMT is a pivotal process that converts epithelial phenotypes to mesenchymal characteristics. Loss of E-cadherin function and increase in N-cadherin expression induces the EMT process, which is involved in tumor metastasis (Thiery and Sleeman, 2006; Kalluri and Weinberg, 2009). To investigate whether G.A could control EMT, we measured mRNA expression levels of EMT-related markers in CRC cells. As shown in **Figure 6A**, low concentration of G.A (5, 10, and 20 μM) treatment for 24 h increased expression levels of E-cadherin in CT26 and HT29 cells, whereas those of N-cadherin and vimentin were reduced in a concentration-dependent manner (**Figures 6B,C**). The expression levels of E-cadherin and N-cadherin were confirmed using a fluorescence microscope. We observed that N-cadherin expression levels decreased in G.A-treated CT26 cells (**Figure 6D**). On the other hand, G.A increased E-cadherin expression in HT29 cells (**Figure 6E**). These results indicate that the EMT of CRC cells was inhibited by G.A treatment.

### Gomisin A Decreases Migration and Invasion of CRC Cells by Inhibiting MMP Activity

**FIGURE 7:**
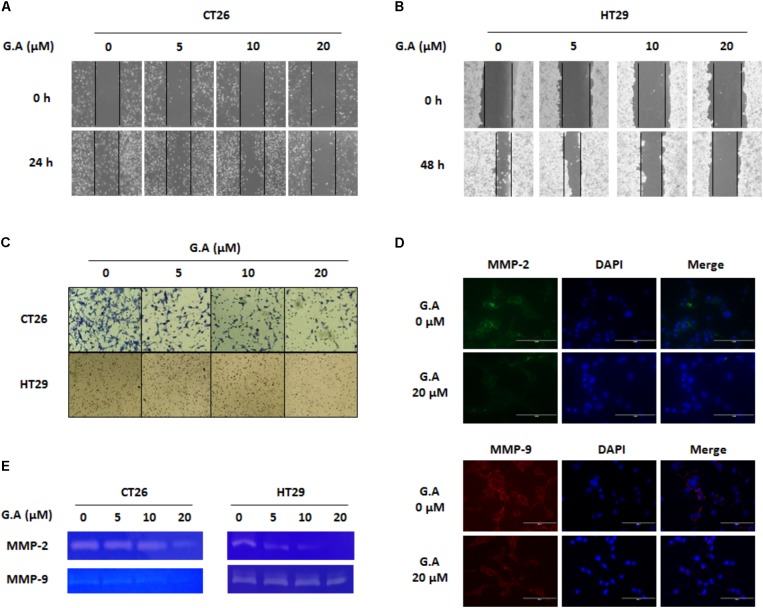
Effect of gomisin A (G.A) on metastatic abilities of CRC cells. CT26 and HT29 cells were treated with G.A (0–20 μM) for 24 h, and metastatic abilities of CRC cells were investigated. **(A,B)** Wound healing assay of G.A-treated CT26 **(A)** and HT29 **(B)** cells. **(C)** Invasion assay. **(D,E)** Matrix metalloproteinase (MMP)-2 and -9 expression levels **(D)** and activity **(E)** of G.A-treated CRC cells. MMP-2 and MMP-9 activity was determined by gelatin zymography. Images were captured using phase contrast microscope (magnification 200×). Data are means ± standard deviation (SD) of three independent experiments.

EMT involves changing the morphological features and gene expression of cancer cells to increase mesenchymal characteristics such as migration and invasion (Martin et al., 2013). Since migration and invasion of cancer cells are necessary for the development of metastasis, wound healing and invasion assays were carried out to evaluate the effect of G.A on these phenomena in CRC cells. Treatment with low concentrations of G.A for 24 h obviously decreased the migration of CT26 and HT29 cells (**Figures 7A,B**). Additionally, the results of the invasion assay showed that the invasion of CT26 and HT29 cells was attenuated by G.A treatment (**Figure 7C**). The gelatinase activity of MMP-2 and MMP-9 is closely related to the invasiveness of cancer cells (Khasigov et al., 2003). We observed that G.A reduced the expression levels of MMP-2 and MMP-9 using IF analysis (**Figure 7D**). Moreover, the results of the gelatin zymography showed that the activity of MMP-2 and MMP-9 concentration-dependently decreased in G.A-treated cells (**Figure 7E**). Thus, G.A reduced the migratory and invasive abilities of CRC cells by suppressing EMT and MMP activity.

## Discussion

Previous studies have reported that G.A is cytotoxic against numerous cancer cell lines including the human LoVo CRC cell line (Smejkal et al., 2010). Furthermore, G.A enhances tumor necrosis factor (TNF)-α-induced G1 cell cycle arrest by downregulating cyclin D1 and retinoblastoma phosphorylation in the HeLa cervical cancer cell line (Waiwut et al., 2012). In this study, G.A reduced the viability of various CRC cell lines by inducing G0/G1 phase arrest and apoptosis. G.A-induced cell cycle arrest is due to downregulation of cyclin D1 and CDK4 expression levels. Apoptosis of CRC cells by G.A treatment was mediated by the cleavage of caspase-3, caspase-9, PARP, and regulation of Bcl-2 family proteins via the AMPK and p38 signaling pathways. Depending on the intensity, extracellular stimuli can damage DNA and induce cell cycle arrest or apoptosis. Although cell cycle arrest is triggered to repair the DNA, excessive damage to DNA leads to apoptosis or necrosis (Shapiro and Harper, 1999; Pucci et al., 2000). In this study, G.A might have damaged the CRC cells and induced cell cycle arrest to repair the DNA. However, considering that TUNEL positive cells were increased by G.A treatment through TUNEL assay, G.A can cause DNA fragmentation in CRC cells. Since G.A at concentrations above 50 μM induces irreparable DNA repair, it consequently promotes apoptosis of CRC cells.

Cellular senescence is permanent cell cycle arrest which is occurred after a finite number of cell division and various extracellular stimuli (Childs et al., 2014). However, cell cycle arrest is not same as senescence because all arrested cells are not senescent cells in the adult organism (Blagosklonny, 2011). Senescent cells show several characteristics including increased senescence-associated β-galactosidase (SA-β-gal) activity and accumulated DNA damage markers such as γ-H2AX (Noren Hooten and Evans, 2017). We performed SA-β-gal staining in G.A-treated cells to confirm whether G.A can induce cellular senescence. As a result, G.A treatment did not occur SA-β-gal activity in both CT26 and HT29 cells. In addition, expression of γ-H2AX was detected in G.A-treated CRC cells. G.A treatment for 24 h did not increase expression of γ-H2AX. Although 20 μM of G.A induced G0/G1 phase arrest but not apoptosis, it was not related to senescence of CRC cells (**Supplementary Figure 3**).

AMPK is a suppressor of cancer progression through the regulation several target proteins and signaling pathway including p53, mTOR, COX-2, and MAPK (Li et al., 2015). Numerous natural products have been reported to show therapeutic potential against several types of cancer, because they can activate AMPK, which inhibits cancer progression (Kim G. T. et al., 2014; Kim M. J. et al., 2014). The MAPKs (ERK, JNK, and p38) signaling pathway is also related to apoptosis of cancer cells (Fang and Richardson, 2005). According to the apoptosis signaling pathway including AMPK, Akt and MAPKs, it has been reported that AMPK activator AICAR can induce apoptosis of leukemia cells by activating Akt (Kuznetsov et al., 2010). AICAR and metformin, which are both AMPK activator, increase apoptosis of pancreatic β-cells through JNK activation (Dai et al., 2015). On the other hand, AMPK activation induces apoptosis by inhibiting phosphorylation of Akt as well as increasing activation of p38 (Cardaci et al., 2012). In the present study, G.A increased phosphorylation of AMPK and p38 (**Figures 5C,D**), but not ERK, JNK, and Akt (**Supplementary Figure 2**). In MCF-7 breast cancer cells, the activation of AMPK by quercetin induces apoptosis through p38 phosphorylation (Kim G. T. et al., 2014). In addition, ginsenoside Rh2 increases the phosphorylation of AMPK and p38, which leads to apoptosis of the HepG2 human hepatoma cells. However, a relationship between AMPK and p38 has not been found (Kim M. J. et al., 2014). We confirmed whether AMPK and p38 are linked in the G.A-induced apoptosis pathway using the specific inhibitors CC and SB203580 (AMPK and p38, respectively). The G.A-induced activation of p38 was inhibited by CC treatment, whereas SB203580 did not affect AMPK phosphorylation. The molecule p38 is downstream of AMPK and, therefore, based on these results, G.A could regulate the survival of CRC cells via the AMPK/p38 signaling pathway.

Furthermore, the antimetastatic effects of G.A on CRC cells were evaluated. G.A inhibited tumor growth and metastasis of B16BL6 cells by suppressing angiogenesis and adhesion of melanoma cells (Kim et al., 2012). Moreover, *S. chinensis* extracts containing G.A also protected against liver metastasis of P815 mastocytoma cells (Tang et al., 2011). In this study, we first demonstrated the inhibitory effects of G.A on the metastasis of CRC cells. EMT, a pivotal step in metastasis, induces the transformation from the epithelial to mesenchymal phenotype that increases migratory and invasive abilities of cancer cells (Thiery and Sleeman, 2006; Kalluri and Weinberg, 2009; Martin et al., 2013). The ethanol extract of *S. chinensis* has been reported to suppress streptozotocin-induced EMT in diabetic nephropathy mice by enhancing E-cadherin expression (Zhang et al., 2012). In general, effect of natural products on the metastatic abilities of cancer cells is determined using non-cytotoxic concentration at proper time course (Liu et al., 2017; Kim et al., 2018). In the present study, effects of G.A on EMT, migration, and invasion of CRC cells were determined using lower concentration of 20 uM, which did not significantly decrease the cell viability of CRC cells after G.A treatment for 24 h. The expression level of the epithelial marker E-cadherin concentration-dependently increased, whereas that of the mesenchymal marker N-cadherin and vimentin were decreased by G.A treatment. IF staining data also demonstrated that G.A upregulated E-cadherin expression, whereas that of N-cadherin was downregulated in CRC cells. As expected, the results of the wound healing and invasion assay also showed that G.A decreased the migratory and invasive ability of CRC cells. These results suggest that G.A could reduce the metastatic characteristics of CRC cells by suppressing the EMT process.

## Conclusion

Gomisin A protected against the metastasis of CRC cells by inhibiting their proliferation and metastatic abilities. G.A induced G0/G1 phase arrest by decreasing cyclin D1 and CDK4 expression levels. Caspases-dependent apoptosis was induced in G.A-treated CRC cells via the AMPK/p38 pathway. Moreover, the migration and invasion of CRC cells were attenuated by inhibiting the EMT process and MMP activity. These findings suggest that G.A might be a potential, novel agent for the prevention and treatment of CRC.

## Author Contributions

J-YK performed all *in vitro* and *in vivo* studies and wrote the manuscript. Y-HH, J-GM, S-HP, and HJ contributed materials and analytical methods. S-HH supervised the study and provided suggestions for the modification of the manuscript.

## Conflict of Interest Statement

The authors declare that the research was conducted in the absence of any commercial or financial relationships that could be construed as a potential conflict of interest.
