# Supplementary material for: Gomisin A Suppresses Colorectal Lung Metastasis by Inducing AMPK/p38-Mediated Apoptosis and Decreasing Metastatic Abilities of Colorectal Cancer Cells
Source: Front Pharmacol. 2018 Aug 29;9:986. doi: 10.3389/fphar.2018.00986 (PMC6124511; doi:10.3389/fphar.2018.00986)
Supplement: Supplementary file 1 [file Table_1.DOC]

**Gomisin A suppresses colorectal lung metastasis by inducing AMPK/p38-mediated apoptosis and decreasing metastatic abilities of colorectal cancer cells**

*Ji-Ye Kee, Yo-Han Han, Jeong-Geon Mun, Seong-Hwan Park, Hee Dong Jeon, and Seung-Heon Hong^*^*

Department of Oriental Pharmacy, College of Pharmacy, Wonkwang-Oriental Medicines Research Institute, Wonkwang University, 460 Iksandae-ro, Iksan, Jeonbuk 54538, Republic of Korea

****Correspondence****:*

Seung-Heon Hong

jooklim@wku.ac.kr

**1. Supplementary Data**

**Senescence-associated β-galactosidase (SA-β-gal) staining**

Senescence β-Galactosidase Staining Kit (Cell Signaling, Danvers, MA, USA) was used to detect SA-β-gal in G.A-treated CRC cells. G.A was treated to CT26 and HT29 cells for 24 h, and SA-β-gal staining was conducted according to the manufacturer’s protocols.

**
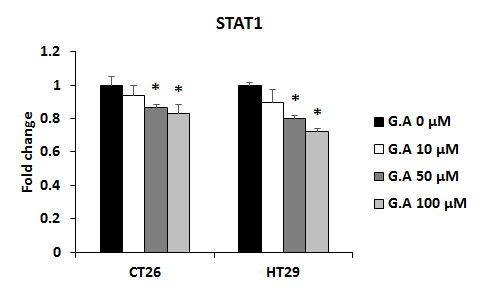
**

**Supplementary Figure 1.** Effect of G.A on the STAT1 expression in CRC cells. G.A was treated to CT26 and HT29 cells for 24 h, and mRNA expression levels were measured by real-time RT-PCR. Data are means ± standard deviation (S.D.) of three independent experiments; **p* < 0.05.


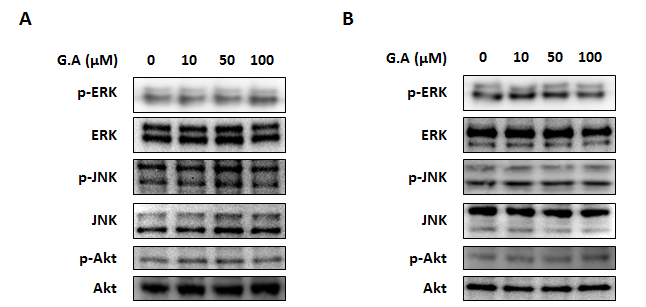


**Supplementary Figure 2.** Effect of G.A on the phosphorylation of ERK, JNK, and Akt in CRC cells. G.A was treated to CT26 (A) and HT29 (B) cells for 24 h, and protein expression levels were detected by western blot analysis.


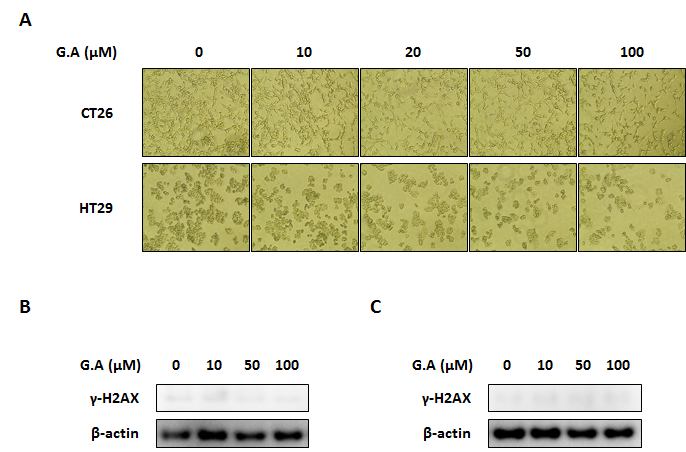


**Supplementary Figure 3.** G.A was treated to CT26 and HT29 cells for 24 h, and senescence-associated β-galactosidase (A) levels were detected by senescence-associated β-galactosidase staining. Expression of γ-H2AX in CT26 (B) and HT29 (C) cells was determined by western blot analysis.
